# Extra-intestinal pathogenic lineages of extended-spectrum β-lactamase (ESBL)-producing *Escherichia coli* are associated with prolonged ESBL gene carriage

**DOI:** 10.1099/acmi.0.000541.v4

**Published:** 2024-02-12

**Authors:** Boas C.L. van der Putten, Jarne M. van Hattem, John Penders, COMBAT Consortium, Daniel R. Mende, Constance Schultsz

**Affiliations:** ^1^​ Department of Medical Microbiology, Amsterdam UMC location University of Amsterdam, Meibergdreef 9, Amsterdam, The Netherlands; ^2^​ Department of Global Health, Amsterdam UMC location University of Amsterdam, Amsterdam Institute for Global Health and Development, Meibergdreef 9, Amsterdam, The Netherlands; ^3^​ School for Public Health and Primary Care (Caphri), Department of Medical Microbiology, Maastricht University Medical Centre, Maastricht, Netherlands; ^4^​ School for Nutrition and Translational Research in Metabolism (NUTRIM), Maastricht University Medical Centre, Maastricht, Netherlands; ^†^​ Present address: Centre for Infectious Diseases Research, Diagnostics and Laboratory Surveillance, National Institute for Public Health and the Environment (RIVM), Bilthoven, Netherlands

**Keywords:** ESBL, *Escherichia coli*, travel, antimicrobial resistance, whole genome sequencing

## Abstract

**Objectives.:**

Extended-spectrum β-lactamase-producing *Escherichia coli* (ESBL-Ec) are frequently acquired during international travel, contributing to the global spread of antimicrobial resistance. Human-adapted ESBL-Ec are predicted to exhibit increased intestinal carriage duration, resulting in a higher likelihood of onward human-to-human transmission. Yet, bacterial determinants of increased carriage duration are unknown. Previous studies analysed small traveller cohorts, with short follow-up times, or did not employ high-resolution molecular typing, and were thus unable to identify bacterial traits associated with long-term carriage after recent acquisition. We aimed to identify which ESBL-Ec lineages are associated with increased carriage duration after return from international travel.

**Methods.:**

In a prospective cohort study of 2001 international travellers, we analysed 160 faecal ESBL-Ec isolates from all 38 travellers who acquired ESBL-Ec during travel and subsequently carried ESBL-Ec for at least 12 months after return, by whole-genome sequencing. For 17 travellers, we confirmed the long-term carriage of ESBL-Ec strains through single nucleotide variant typing. To identify determinants of increased carriage duration, we compared the 17 long-term carriers (≥12 months of carriage) with 33 age-, sex- and destination-matched short-term carriers (<1 month of carriage). Long-read sequencing was employed to investigate long-term ESBL plasmid carriage.

**Results.:**

We show that in healthy travellers with very low antibiotic usage, extra-intestinal pathogenic lineages of *E. coli* (ExPEC) are significantly more likely to persist than other *E. coli* lineages. The long-term carriage of *E. coli* from ExPEC lineages is mainly driven by sequence type 131 and phylogroup D *E. coli*.

**Conclusions.:**

Although ExPEC lineages frequently cause extra-intestinal infections such as bloodstream infections, our results indicate that ExPEC lineages are also efficient intestinal colonizers, which potentially contributes to their onward transmission.

## Data Summary

Whole-genome sequencing reads (Illumina and Oxford Nanopore Technologies) are deposited in NCBI BioProject PRJEB40103. Version 1.1.0 of the bioinformatics code and metadata is archived through Zenodo (https://doi.org/10.5281/zenodo.4582689). Previous versions are available from https://www.github.com/boasvdp/COMBAT. Consortium^#^

**Impact Statement**

Resistant bacteria, such as *Escherichia coli* expressing extended-spectrum β-lactamase enzymes (ESBL-Ec), are readily acquired during international travel. The resulting international spread may contribute to an increase of antimicrobial resistance in low-prevalence countries. We analysed acquisition and carriage of ESBL-Ec in a large traveller cohort using whole-genome sequencing. Due to the unprecedented sample size of the study (2001 travellers), we were able to compare age-, sex- and destination-matched short-term carriers to long-term (≥12 months) carriers, allowing us to identify ESBL-Ec sequence types which are significantly associated with prolonged carriage after acquisition during travel. Our results advance our understanding of the adaptation to the human intestinal environment by ESBL-Ec and pinpoint ESBL-Ec sequence types that should be monitored to prevent increases in antimicrobial resistance carriage and onward spread.

## Introduction

**Fig. 1.**
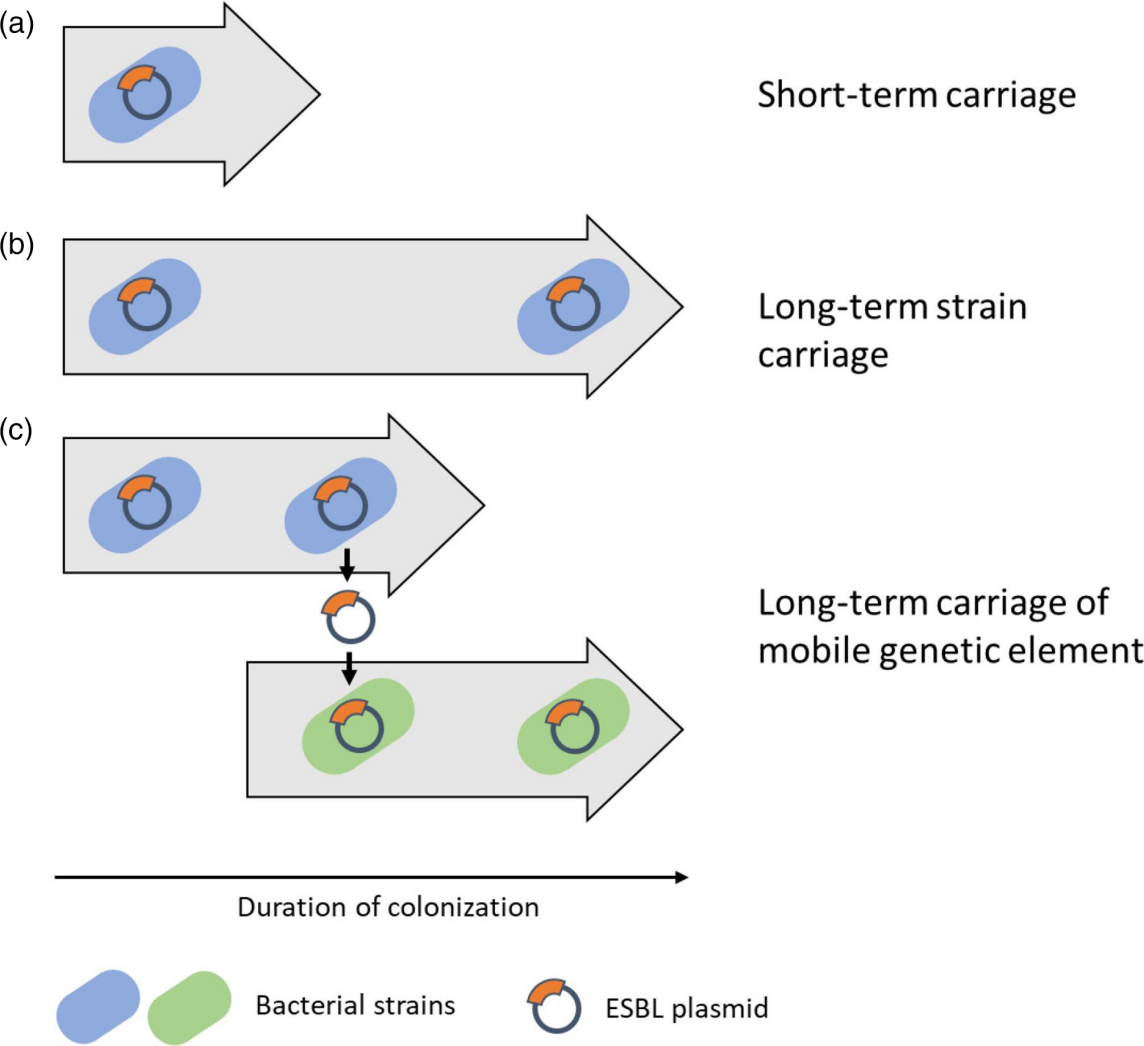
Schematic overview of main long-term carriage mechanism of ESBL genes in the gut of returning travellers. Grey arrows indicate carriage duration over time. (**a**) Short-term carriage, where the colonizing strain is lost during the follow-up period. (**b**) Long-term strain carriage, where a bacterial strain harbouring an ESBL gene is continuously present. (**c**) long-term carriage of mobile genetic elements, where the original strain harbouring the ESBL gene has been lost, but the ESBL gene (located on a mobile genetic element) has been passed on to another strain.

International travel contributes significantly to the spread of extended-spectrum β-lactamase (ESBL) gene-positive *Escherichia coli* (ESBL-Ec) [1, 2]. Genetically diverse ESBL-Ec are frequently acquired during international travel [3–5]. Whilst travel-acquired ESBL-Ec typically are lost during travel or within the first month after return (Fig. 1a) [6], ESBL-Ec and ESBL genes have been detected for more than 12 months after return in a proportion of travellers [1, 3–5, 7]. Additionally, several European and North America studies found that recent international travel is a risk factor for ESBL carriage [8–15]. A recent Dutch study reported an adjusted odds ratio of 3.16 [95 % confidence inteval (CI): 1.71–5.83] [8], while a meta-analysis of international studies reported a risk ratio of 4.06 (95 % CI: 1.3–12.41) [15]. At least two mechanisms are possible for the persistence of ESBL genes [16]. Long-term strain carriage (Fig. 1b) is the first mechanism, where a bacterial strain carrying an ESBL gene persists within the local microbiome. A second mechanism is long-term carriage of a mobile genetic element (Fig. 1c), where an ESBL gene located on, for example, a mobile plasmid persists in the local microbiome, by transferring between multiple transiently colonizing strains.

The likeliness of onward transmission of ESBL genes within an environment is probably dependent on how long a strain persists within that environment. Thus, ESBL genes carried by strains that persistently colonize the human gut microbiome are likely to have a higher chance of onward transmission than ESBL genes harboured by transiently colonizing strains. Therefore, it is important to understand which ESBL-harbouring lineages persist most efficiently after acquisition during international travel. Elucidating this has proven challenging, as a prospective study design, a sufficiently large sample size and the use of high-resolution typing methods such as whole-genome sequencing (WGS) are needed to determine which lineages persist for an extended period of time.

Several previous studies either did not include WGS or followed travellers for a short duration after return from travel and will not be discussed in detail here [4, 5]. A recent study demonstrated that of 16 travellers who acquired ESBL-Ec abroad, only a single traveller carried the acquired ESBL-Ec strain for at least 7 months after return [7]. Another recent study included data from 11 travellers who carried ESBL-Ec for at least 3 months [17]. Few ESBL-Ec attributes could be associated with long-term carriage in either study, partially due to low sample size. Armand-Lefèvre *et al*. reported an association of three phylogroups (B2/D/F) with increased carriage duration (*P*=0.02) [17]. Notably, extra-intestinal pathogenic *E. coli* (ExPEC) strains are often identified among these particular phylogroups. ExPEC are *E. coli* types often isolated from extra-intestinal disease manifestations such as bloodstream infections, urinary tract infections or meningitis [18]. Many ExPEC strains are genetically related and cluster together in a limited number of sequence types (STs) such as ST69, ST73 and ST131 (‘ExPEC lineages’) [19]. It has been postulated previously that these ExPEC lineages display an enhanced capability of intestinal colonization [20]. In a recent study, no single ExPEC gut colonization event during travel could be linked to extra-intestinal infections [21]. This suggests that although these strains are capable of causing extra-intestinal disease, they are not always pathogenic [18]. In this study, we aimed to identify ESBL-Ec lineages which are associated with an increased carriage duration in healthy travellers after they returned from international travel.

## Methods

### Travellers and isolate selection

**Fig. 2.**
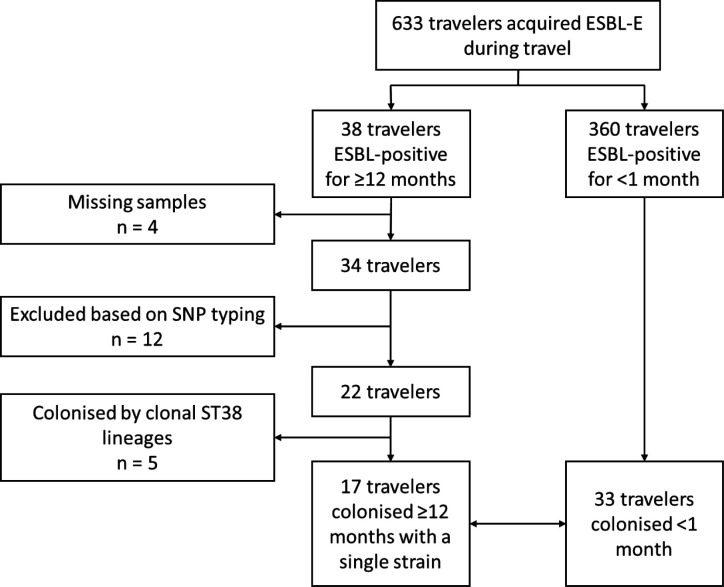
Study flowchart. Long-term strain carriage was defined as isolates from a single traveller, sampled 12 months apart with identical ESBL alleles and 25 or fewer genome-wide SNP difference. Clonal ST38 isolates were excluded. Seventeen long-term carriers (colonized for ≥12 months with a single strain) were matched on sex, age and travel destination to 33 short-term carriers (colonized for <1 month).

Of 2001 international travellers included in the COMBAT cohort, 633 acquired ESBL-E abroad (Fig. S1, available in the online version of this article). A total of 38 travellers were considered potential long-term ESBL-positive *E. coli* carriers, based on epidemiological and PCR data. These travellers fulfilled both of the following criteria: (1) no ESBL gene was detected before travel; and (2) at return from travel and 1, 3, 6 and 12 months after return, the traveller tested positive for the same ESBL gene group. Arcilla *et al*. provide a more detailed description of sampling and microbiology [1]. No travellers were long-term carriers of ESBL-E other than *E. coli*, which is why we have focused exclusively on ESBL-Ec in this paper. In short, faecal samples were inoculated overnight in tryptic soy broth supplemented with 50 mg l^−1^ vancomycin to enrich for *Enterobacterales*. The broth was subsequently cultured on chromID ESBL agar plates (bioMérieux). One to five isolates per faecal sample were selected (median: one isolate per sample). If multiple morphologies were observed, these were separately isolated. For the current study a total of 160 ESBL-Ec isolates were selected (Fig. 2), of which 85 were sampled at return from travel (termed T0) and the remaining 75 isolates were sampled 12 months after return from travel (termed T12).

### Bioinformatic analysis

DNA was extracted using the Qiagen Blood and Tissue DNA extraction kit (ID: 69506). Sequencing libraries were prepared using the Kapa HTP library prep kit (ID: KK8234). The Amsterdam UMC Core Facility Genomics performed WGS on the Illumina HiSeq 2500 platform, producing paired-end reads of 150 bp.

The full workflow is archived at https://doi.org/10.5281/zenodo.4582689. Snakemake v5.7.1 was used to manage analyses [22] and unless, noted otherwise, default settings were used. The program fastp v0.20.0 was used to trim and filter sequence reads [23], and Shovill v1.0.9 (https://github.com/tseemann/shovill) was used to assemble cleaned reads using SPAdes [24]. AMRfinderplus v3.2.3 was used to detect antimicrobial resistance (AMR)-associated genes and mutations [25]. EzClermont v0.4.3 was used to predict phylogroups [26]. The program mlst (https://github.com/tseemann/mlst), using the Achtman scheme [27], was used to identify multi-locus sequence types from assembled draft genomes. Finally, ABRicate v1.0.1 (https://github.com/tseemann/abricate) was used to identify plasmid replicons and virulence-associated genes using the PlasmidFinder [28] and VFDB [29] databases, respectively.

### Analysis of long-term strain carriage

In the current study, we designate travellers as long-term strain carriers if the traveller carries the same ESBL-Ec strain at return from travel (T0) and 12 months thereafter (T12). We consider two isolates belonging to the same strain if they differ in a maximum of 25 SNP positions, normalized to an approximate genome length of 5 million base pairs [30].

To assess SNP differences, we used Snippy v4.4.5 (https://github.com/tseemann/snippy) to map cleaned reads on appropriate reference genomes. We used ReferenceSeeker v1.6.3 [31] to identify appropriate reference genome per multilocus sequence type (MLST). Selected reference genomes and genome alignment lengths are available in Table S1. IQtree v1.6.12 [32] was used to infer a phylogeny for each alignment, using Modelfinder to automatically select an appropriate model [33]. ClonalFrameML v1.12 [34] was used to detect recombination events in the alignments, which were subsequently masked using maskrc-svg v0.5 (https://github.com/kwongj/maskrc-svg). A modified version of snp-dists v0.7.0 (https://github.com/boasvdp/snp-dists) was used to count SNP differences and alignment lengths. To approximate the number of genome-wide SNPs, the absolute number of SNPs was normalized to an approximately normal genome length of 5 Mb [30].

We generated Oxford Nanopore Technologies (ONT) sequencing data according to Van der Putten *et al*. [35]. Isolates were grown in liquid LB at 37 °C, overnight. The Qiagen MagAttract HMW DNA Kit (ID: 67563) was used to extract high-molecular-weight DNA. ONT sequencing libraries were prepared using the ONT native barcoding kit (EXP-NBD114). An ONT MinION flowcell was used to sequence the prepared library. Filtlong v0.2.0 (https://github.com/rrwick/Filtlong) was used to trim and filter the resulting sequence reads. Unicycler v0.4.8 [36] was employed to assemble long- and short-read data together. FastQC v0.11.8 [37], Quast v4.6.3 [38] and MultiQC v1.6 [39] were included for intermediate quality control. ANICalculator [40] was used to calculate the extent of similarity between assembled plasmids.

### Comparison with short-term carriers

Using SPSS 26, we attempted to match every long-term carrying traveller based on age, sex and travel destination to two short-term carrying travellers (Fig. 2). For all 41 ESBL-Ec isolates sampled from matched short-term carriers, we performed Illumina WGS as described above.

### Plotting and statistical analysis

Tbe packages ggplot2 v3.1.1 [41], ggthemes v2.4.0 [42], and patchwork [43] in R v3.5.1 were used for data visualization. Pandas v0.24.2 [44] in Python v3.6.7 was used for data analysis. For comparison of groups we used Fisher’s exact test (implemented in SciPy) [45].

## Results

Of all 38 travellers who were considered potential long-term carriers based on epidemiological and PCR data, we were able to include 34 travellers with available samples (Fig. 2) [1]. We refer to the original publications of the COMBAT study for more details on inclusion of travellers [1, 46].

### Long-term strain carriage of ESBL-Ec

#### SNP analysis

Of 34 included travellers, 22 harboured the same strain for at least 12 months after return from travel, based on SNP analysis (≤25 SNPs in a 5 Mb alignment, Fig. 2). For 12 other travellers, we did not identify the same strain at return from travel and 12 months thereafter, based on SNP analysis. Of these 12 remaining travellers, 10 harboured strains belonging to different STs at return from travel and 12 months thereafter. The remaining two travellers harboured strains from the same ST, but these differed more than the specified 25 SNPs per 5 Mb alignment [30]. While most travellers acquired a single ESBL-Ec strain, we also observed travellers who acquired up to four distinct strains at a single timepoint.

#### Clonal sequence type 38 lineages

To analyse the genetic diversity of ESBL-Ec in general, we also compared isolates between unrelated travellers (e.g. from different households). Here, we observed two ESBL-Ec lineages belonging to ST38 to be common among travellers. Per lineage, these isolates were closely related to other isolates within the same lineage, even showing fewer SNP differences than the clonality threshold used in this paper (25 SNPs per 5 Mb alignment).

Lineage ST38-*bla*_CTX-M-27_ was identified in three (unrelated) travellers and lineage ST38-*bla*_CTX-M-14_ in two other travellers. These five travellers returned from different continents and/or countries, suggesting they did not acquire these ST38 strains from a single source during travel. Lineages ST38-*bla*_CTX-M-27_ and ST38-*bla*_CTX-M-14_ were also identified in public sequence repositories. Based on these public data, we confirmed that these lineages are highly clonal (Fig. S2). Because these lineages often appeared in our dataset with SNP differences below our clonality threshold, even though the isolates were sampled from unrelated travellers, we concluded we would not be able to distinguish between long-term strain carriage or reacquisition of these clonal lineages. Thus, we excluded travellers who harboured the clonal ST38-*bla*_CTX-M-27_ and ST38-*bla*_CTX-M-14_ lineages from further analysis (Fig. 2). Four travellers harbouring other ST38 lineages were not excluded, as these lineages did not display such a level of clonality.

Two travellers, belonging to the same household, harboured ST69 isolates which only differed by 9 SNPs. This strain was included for further analysis. Based on available data, we could not distinguish whether this strain was acquired by one traveller from the other traveller, or whether both travellers acquired the strain from a single source.

#### ESBL plasmid analysis

A total of six travellers were included for ESBL plasmid analysis. These travellers harboured identical ESBL genes at return from travel and 12 months after return from travel, but SNP analysis showed that the ESBL genes were present in different bacterial strains, when comparing these timepoints (Table S2). ESBL plasmid analysis was performed by combining long-read ONT sequencing with the available short-read Illumina sequencing. A single traveller harboured a highly similar ESBL plasmid (99 % of the plasmid aligned with 99.8 % nucleotide identity) at return from travel and 12 months thereafter, while the plasmid was present in ESBL-Ec belonging to different STs. This suggested long-term ESBL gene carriage through plasmid persistence (Fig. 1c). The persistent plasmid was 87 kb long, harboured a single IncB/O/K/Z replicon and had *bla*_CTX-M-14_ as the only resistance gene. The remaining travellers harboured dissimilar plasmids, suggesting these travellers had probably reacquired the same ESBL gene but in different plasmid backgrounds and different bacterial strains.

In summary, we identified long-term carried ESBL-Ec strains in 17 of 34 travellers with prolonged ESBL-Ec carriage and a long-term carried ESBL-plasmid in one traveller (Table S2). Although we included multiple ESBL-Ec isolates for some travellers, we did not observe multiple long-term carried strains within any single traveller.

### Comparison of ESBL-Ec from long- and short-term carriers

For each long-term carrier (≥12 months of carriage) harbouring a long-term carried strain, two short-term carriers (<1 month of carriage) were matched by age, sex and travel destination. For one long-term carrier, only a single matching short-term carrier could be identified, resulting in a comparison of 17 isolates from 17 long-term carriers and 42 isolates from 33 matched short-term carriers, which were sequenced (Table 1).

**Table 1.** Comparison of isolates of long-term (≥12 months) and short-term (<1 month) carriers Visited countries are sorted alphabetically per traveller. ExPEC lineage status is based on ExPEC lineage definition by Manges *et al*. [19]. A dash in MLST columns indicates no MLST could be assigned. Matching on travel destination was done by United Nations subregions. MAR=months after return. MLST=multilocus sequence type. T1=Timepoint 1 (within 1 month upon return from travel). T12=Timepoint 12 (12 months after return from travel). UAE=United Arab Emirates.

Long-term carrier	Countries visited	Antibiotic use	Isolate T1	Isolate T12	MLST	Phylogroup	ExPEC lineage	Matched short-term carriers	Countries visited	Antibiotic use	Isolate T1	MLST	Phylogroup	ExPEC lineage
trav007	China, Nepal	No	isol058	isol170	393	D	Yes	trav008	Sri Lanka, UAE	Unknown antibiotic (before travel), unknown antibiotic (6–12 MAR)	isol056	–	Cryptic	No
								trav087	India	No	isol143	–	D	No
trav010	Cambodia, Laos, Thailand, Vietnam	No	isol120	isol192	393	D	Yes	trav019	Cambodia, Vietnam	No	isol151	226	A	No
											isol152	226	A	No
								trav077	Indonesia	Nitrofurantoin (3–6 MAR)	isol106	3171	A	No
											isol107	609	A	No
trav011	Thailand	Ciprofloxacin (during travel)	isol094	isol187	405	D	Yes	trav027	Thailand	No	isol016	542	A	No
											isol017	48	A	No
								trav036	Indonesia	Doxycyclin (3–6 MAR)	isol028	205	B1	No
trav030	Indonesia	Ciprofloxacin (6–12 MAR)	isol012	isol158	131	B2	Yes	trav020	Cambodia	No	isol146	38	D	Yes
								trav073	Malaysia, Singapore	No	isol100	38	D	Yes
trav032	India	No	isol202	isol235	10	A	Yes	trav035	Bangladesh, India	No	isol015	–	A	No
								trav075	India	No	isol102	226	A	No
trav040	Thailand	No	isol030	isol164	131	B2	Yes	trav014	Indonesia	Metronidazol (1–3 MAR)	isol116	3171	A	No
								trav066	Indonesia	Unknown antibiotic (1–3, 3–6 and 6–12 MAR)	isol112	7122	A	No
trav044	India	No	isol043	isol169	38	D	Yes	trav005	India	No	isol039	–	A	No
								trav038	India	No	isol031	167	A	Yes
											isol032	648	F	Yes
trav046	China	No	isol077	isol177	69	D	Yes	trav056	China	Unknown antibiotic (6–12 MAR)	isol061	1434	A	No
								trav061	China, Mongolia	No	isol084	162	B1	No
trav047	China	No	isol079	isol176	69	D	Yes	trav099	China	No	isol104	1193	B2	Yes
								trav103	China	No	isol069	2346	B2	No
trav051	Peru	No	isol052	isol174	449	D	No	trav084	Bolivia, Peru	No	isol134	88	C	Yes
								trav106	French Guiana, Surinam	No	isol136	6488	B1	No
trav064	Thailand	Unknown antibiotic (6–12 MAR)	isol076	isol182	38	D	Yes	trav003	India, Malaysia, Thailand, UAE	Ciprofloxacin (during travel)	isol051	443	B1	No
								trav111	Indonesia	No	isol114	48	A	No
											isol115	381	B1	No
trav076	Vietnam	Ciprofloxacin and trimethoprim (during travel)	isol109	isol186	131	B2	Yes	trav016	Myanmar, Thailand, UAE	Pheneticillin (during travel), unknown antibiotic (6–12 MAR)	isol145	1193	B2	Yes
								trav088	Laos, Thailand	No	isol133	3171	A	No
trav079	China, India, Nepal	Clindamycin (6–12 MAR)	isol119	isol191	38	D	Yes	trav080	Nepal	No	isol118	58	B1	Yes
								trav109	India, Nepal	No	isol022	602	B1	No
											isol023	227	A	No
trav086	China	No	isol139	isol194	405	D	Yes	trav104	China	No	isol068	744	A	No
								n/a	n/a	n/a	n/a	n/a	n/a	n/a
trav093	Nepal	Unknown antibiotic (1–3 MAR)	isol062	isol175	38	D	Yes	trav101	India	Unknown antibiotic (0–1, 1–3, 3–6 and 6–12 MAR)	isol150	70	D	No
								trav102	Nepal	Unknown antibiotic (1–3, 3–6 and 6–12 MAR)	isol060	3910	E	No
trav095	Sri Lanka	Unknown antibiotic (during travel)	isol087	isol181	131	B2	Yes	trav023	India	Unknown antibiotic (0–1, 1–3, 3–6 and 6–12 MAR)	isol011	1312	cryptic	No
								trav078	India	Unknown antibiotic (0–1, 3–6 and 6–12 MAR)	isol122	485	E	No
											isol123	227	A	No
											isol124	485	E	No
trav098	India	No	isol090	isol184	–	A	No	trav070	China, Nepal	No	isol099	155	B1	No
								trav107	Nepal	No	isol121	398	D	No

Antibiotic usage was low before, during and after travel and was similar between long-term and short-term carriers (Table 2). None of the travellers were admitted to the hospital during or after travel in either group. One single traveller returned to the same country as visited during index travel, within 12 months after return from index travel.

**Table 2.** Characteristics of long-term and short-term carriers Matching was performed on sex, age and travel destination (United Nations subregions). These characteristics are depicted in italic. Data are presented as number (%) for all characteristics except age and travel duration, which are presented as median (IQR).

		Long-term carriers (*n*=17)	Short-term carriers (*n*=33)
*Male*		4 (23.5 %)	8 (24.2 %)
*Age (years)*		50.2 (41.7–59.4)	51.4 (36.5–58.1)
*Continent visited during index travel*	*Asia*	16 (94.1 %)	31 (93.9 %)
	*North and South America*	1 (5.9 %)	2 (6.1 %)
Travel duration (days)		20 (17–28)	19 (14–21)
Admitted to hospital during index travel		0 (0 %)	0 (0 %)
Travel to the same country as index travel, within 12 months after return from index travel		1 (5.9 %)	0 (0 %)
Antibiotic usage	Within 3 months before index travel	0 (0 %)	0 (0 %)
	During index travel	3 (17.6 %)	2 (6.1 %)
	Within 1 month after return from index travel	0 (0 %)	0 (0 %)
	Within 1–3 months after return from index travel	1 (5.9 %)	2 (6.7 %)
	Within 3–6 months after return from index travel	0 (0 %)	2 (7.1 %)
	Within 6–12 months after return from index travel	3 (17.6 %)	2 (7.4 %)
Traveller’s diarrhoea		6 (35.3 %)	9 (27.3 %)

Using a recently proposed definition of ExPEC lineages [19], we found that long-term strain carriage was significantly associated with isolates belonging to ExPEC lineages. Strains belonging to ExPEC lineages persisted in 15 out of 17 long-term carriers, while ExPEC lineages were sampled from only seven out of 33 short-term carriers (odds ratio 27.86, 95 % CI: 5.11–151.74). The main ExPEC lineages observed were ST131 and STs belonging to phylogroup D.

Among 17 strains from long-term carriers and 41 strains from short-term carriers, *bla*_CTX-M-15_ was the most common ESBL gene (*n*=29, 50 %), followed by *bla*_CTX-M-14_ (*n*=9, 16 %). The pandemic ST131 lineage was observed four times, with three different *bla*_CTX-M_ genes: *bla*_CTX-M-15_ (*n*=2), *bla*_CTX-M-14_ and *bla*_CTX-M-27_ (both *n*=1). The triple mutation of *gyrA* Ser80Leu, *gyrA* Asp87Asn and *parC* Ser80Ile, which together predicts clinical resistance to ciprofloxacin [47], was found in 25 isolates (43 %) and among 14 distinct STs, including ExPEC lineages ST10, ST38, ST131, ST393, ST405, ST648 and ST1193. Common virulence-associated factors were present in nearly all isolates, such as type I fimbriae (encoded by *fim* genes) and enterobactin (encoded by *ent*, *fep* and *fes* genes). Other siderophores were also present such as aerobactin in 25 isolates (42 %, encoded by *iucABCD-iutA*), and yersiniabactin in 30 isolates (51 %, encoded by *fyuA*, *irp1, irp2* and *ybtAEPQSTUX* genes). Some virulence-associated or resistance-associated genes were only found in low numbers of isolates: four isolates (7 %) harboured *iroBCDEN* (encoding the salmochelin siderophore), three isolates (5 %) harboured *kpsDMT* (encoding a K1 capsule) and three isolates (5 %) harboured *hlyABCD* (encoding haemolysin). Full operons of *sfa* or *pap* (encoding S fimbriae and P fimbriae, respectively) were not observed. No carbapenemase-encoding or *mcr* genes were observed.

## Discussion

Out of 2001 international travellers, we identified 17 travellers who carried the same ESBL-Ec strain for at least a year after return. The long-term carriage was mainly driven by ExPEC lineages, more specifically ST131 and phylogroup D. For another traveller, our data suggest that an ESBL plasmid has persisted for 12 months upon return from travel, and that the plasmid has shifted between bacterial hosts.

Our findings support previous conclusions that the lineage of acquired ESBL-Ec influences carriage duration upon return from travel [17]. Due to a larger sample size, we could link long-term carriage to specific ExPEC STs, arriving at similar conclusions as Armand-Lefèvre *et al.*, who concluded that three broad *E. coli* phylogroups (B2, D and F) displayed increased carriage duration.

Other previous studies could not reach firm conclusions on long-term ESBL-Ec carriage after return from travel, either due to smaller sample sizes, shorter follow-up periods, the lack of high-resolution typing (e.g. WGS) or because the study population did not represent exposure observed in the community [3–5, 7]. One recent study performed very intensive sampling of a group of travellers to Laos, which showed that a high diversity of ESBL-Ec strains was acquired, and that acquisition and loss of ESBL-Ec is highly dynamic [6]. It should be noted that these travellers attended a course which included multiple visits to local hospitals which might have increased the odds of acquiring ESBL-Ec.

ExPEC often colonize the human gut microbiome without causing intestinal symptoms [18, 48]. It has been suggested that the pathogenicity of ExPEC outside the intestine is an evolutionary ‘byproduct’, as the evolution of ExPEC was driven by selective pressure towards efficient gut colonization [20]. Our results underline the notion that ExPEC lineages are spreading globally due to efficient gut colonization. Long-term colonized travellers harbour ExPEC for up to a year after return, even though these travellers are exposed to ExPEC abroad for only a short duration in our study (median 20 days, interquartile range: 17–29 days). However, we focused solely on ESBL-Ec in our study, so we cannot make assertions on the persistence of non-ESBL-encoding ExPEC based on our results. Since only 2.7 % (17 out of 633) of travellers who acquired ESBL-E during travel carried the acquired ESBL-E for more than 12 months, our sample size was limited. Had we been able to include more travellers, we would have investigated which genetic elements specifically are associated with long-term carriage. However, large longitudinal cohorts are challenging and expensive to initiate and maintain. The current best predictor, ExPEC lineage membership, is strongly associated with the presence of virulence or colonization factors due to population structure, complicating the exact determination of genes driving long-term carriage.

We detected two highly clonal lineages of ST38, which were shared between unrelated travellers. These lineages were also abundant in public data from global sources, and showed a similar level of clonality. The clonality of these lineages hindered our genomic analysis of strain carriage. Possibly, future methodological advances could help in correctly analysing strain carriage in cases such as these. Current developments include software applying SNP analysis to the pangenome, instead of to a single fixed reference genome [49]. Although the error rate can be higher than traditional methods, these new advances are a promising avenue for future research [49]. The possibility that certain lineages are able to spread clonally is important to consider, for example when analysing suspected outbreaks of ST38.

Even though our study only considered ESBL-Ec, the general methodology can also be used to analyse other bacterial carriage studies. An interesting possibility would be to assess whether among non-resistant *E. coli*, ExPEC lineages are also driving long-term strain carriage. If this is to be attempted, it should be considered to increase the frequency of sampling, as the current study employed a sparsely sampled follow-up period. However, when comparing between two timepoints, 12 months apart, we could still identify very closely related isolates in samples from the same traveller, indicating long-term strain carriage. Additionally, we only had isolates available for household members of four long-term carriers. Including more household members in future studies might allow us to estimate the likelihood of onward transmission following long-term carriage.

Applying genomic epidemiology to a large traveller cohort, we have shown that ESBL-positive *E. coli* acquired during travel are able to colonize the traveller’s gut for more than a year. The strains that showed the longest carriage duration belonged predominantly to pathogenic ExPEC lineages. Our data imply that long-term carriage of resistant *E. coli* is governed by bacterial characteristics which are associated with lineage. This finding possibly allows a more precise risk assessment for international travellers returning with travel-acquired resistant *E. coli*.

## Supplementary Data

Supplementary material 1

Supplementary material 2
