# Supplementary material for: Extra-intestinal pathogenic lineages of extended-spectrum β-lactamase (ESBL)-producing Escherichia coli are associated with prolonged ESBL gene carriage
Source: Access Microbiol. 2024 Feb 12;6(2):000541.v4. doi: 10.1099/acmi.0.000541.v4 (PMC10928385; doi:10.1099/acmi.0.000541.v4)
Supplement: Supplementary material 1 [file acmi-6-541.v4-s001.pdf]

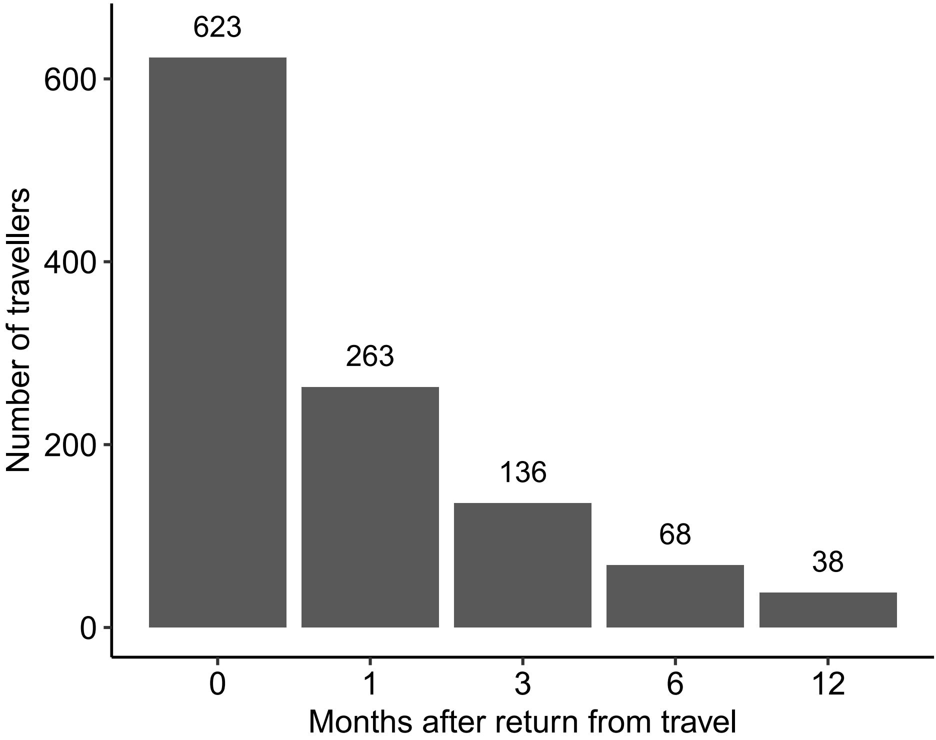

Figure S1. Bar plot showing the number of travelers for which the same ESBL gene group was detected in every sample up until that timepoint. A total of 633 travelers acquired ESBL-E of which 38 travelers were still positive for the same ESBL gene group at all timepoints.

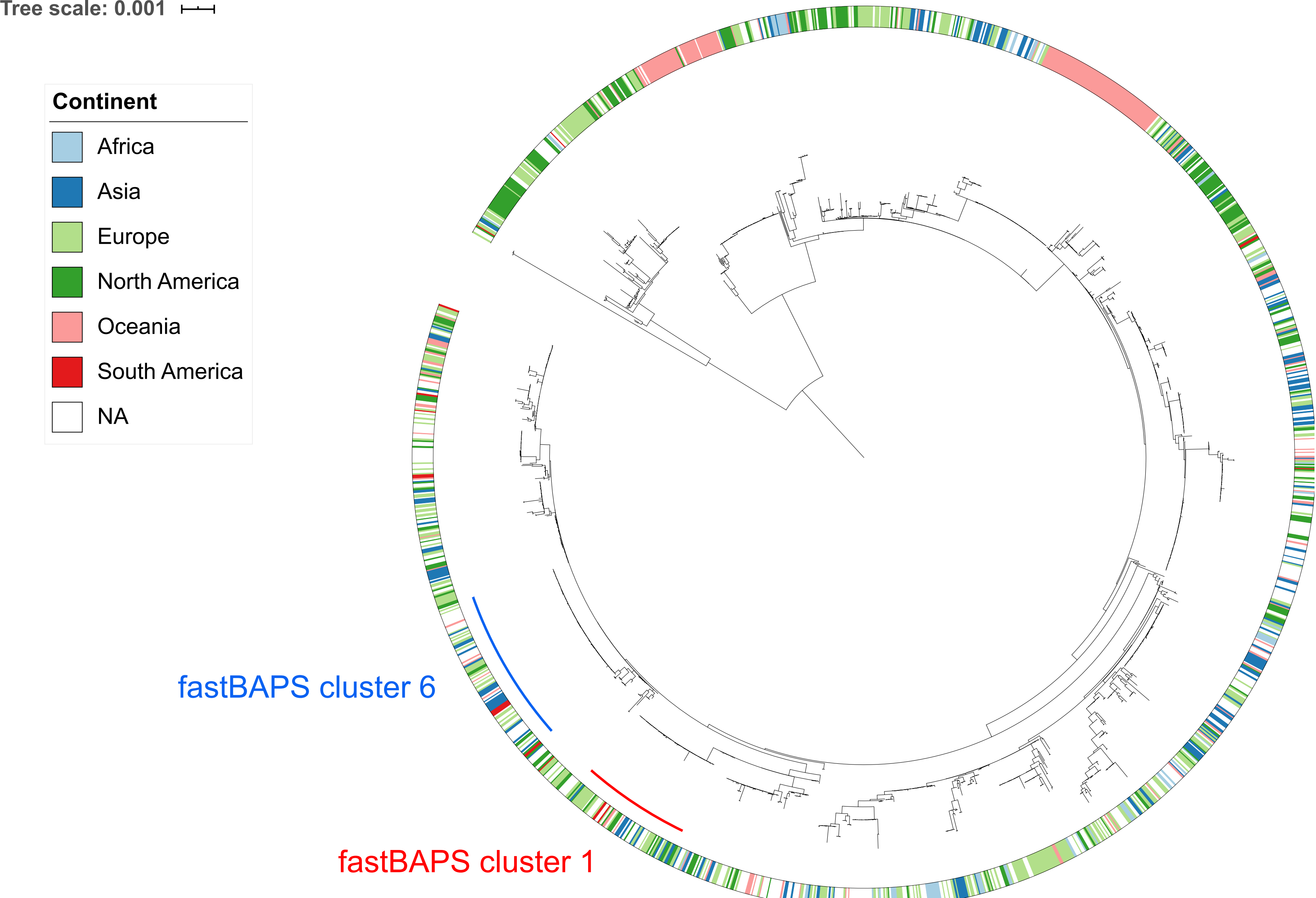

Figure S2. Core genome phylogeny of 1805 clonal complex 38 *E. coli* strains. Outer ring indicates continent on which the strain was isolated. The two clonal ST38 lineages present in the COMBAT collection are marked in red and blue. Tree and metadata available through iTOL: https://itol.embl.de/tree/2131278347268071589210657.

The members of the Carriage Of Multiresistant Bacteria After Travel (COMBAT) consortium, in alphabetical order: Maris S. Arcilla, Martin C.J. Bootsma, Perry J. van Genderen, Abraham Goorhuis, Martin Grobusch, Jarne M. van Hattem, Menno D. de Jong, Damian C. Melles, Nicky Molhoek, Astrid M.L. Oude Lashof, John Penders, Constance Schultsz, Ellen E. Stobberingh, Henri A. Verbrugh.
